# Galectin-1 as the new player in staging and prognosis of COVID-19

**DOI:** 10.1038/s41598-021-04602-z

**Published:** 2022-01-24

**Authors:** Sofija Sekulic Markovic, Nevena Gajovic, Milena Jurisevic, Marina Jovanovic, Biljana Popovska Jovicic, Nebojsa Arsenijevic, Zeljko Mijailovic, Marina Jovanovic, Zana Dolicanin, Ivan Jovanovic

**Affiliations:** 1 Department of Infectious Disease, Faculty of Medical Sciences, University of Kragujevac, 34000 Kragujevac, Serbia; 2 Faculty of Medical Sciences, Center for Molecular Medicine and Stem Cell Research, University of Kragujevac, 34000 Kragujevac, Serbia; 3 Department of Clinical Pharmacy, Faculty of Medical Sciences, University of Kragujevac, 34000 Kragujevac, Serbia; 4 Department of Internal Medicine, Faculty of Medical Sciences, University of Kragujevac, Svetozara Markovica 69, 34000 Kragujevac, Serbia; 5 Public Health Institute Kragujevac, 34000 Kragujevac, Serbia; 6 Department of Otorinolaringology, Faculty of Medical Sciences, University of Kragujevac, 34000 Kragujevac, Serbia; 7 Department of Biomedical Sciences, State University of Novi Pazar, 36300 Novi Pazar, Serbia

**Keywords:** Immunology, Diseases

## Abstract

A new virus from the group of coronaviruses was identified as the cause of atypical pneumonia and called Severe Acute Respiratory Syndrome Coronavirus-2 (SARS-CoV-2) and disease called Corona Virus Disease (COVID-19). During the cytokine storm, the main cause of the death, proinflammatory cytokines are released which stimulate further tissue destruction. Galectin-1 (Gal-1) is a pleiotropic cytokine involved in many immune and inflammatory processes and its role in COVID-19 is still unknown. The aim of this study was to determine systemic values of Gal-1 and correlations between Gal-1 and proinflammatory cytokines and clinical parameters during COVID-19 progression. This is observational and cross-sectional study. 210 COVID-19 patients were included and divided into mild, severe or critical group according to COVID-19 severity. Serum levels of IL-1β, IL-6, IL-10, IL-23, IL-33 and Gal-1 were measured using sensitive enzyme-linked immunosorbent assay (ELISA) kits. Systemic levels of IL-1β, IL-6, IL-10, IL-23, IL-33 and Gal-1 were significantly higher in stage III of COVID-19 patients compared to stage I and II. There were no significant differences in the ratio between Gal-1 and IL-10 with proinflammatory cytokines. Positive correlation was detected between Gal-1 and IL-1β, IL6, IL-10, IL-23 and IL-33. Gal-1 positively correlated with chest radiographic finding, dry cough and headache and negatively correlated with normal breathing sound. Linear regression model and ROC curve analysis point on Gal-1 as significant predictor for COVID-19 severity. Presented results implicate on Gal-1 and IL-10 dependent immunomodulation. The precise mechanism of Gal-1 effect in COVID-19 and its potential as a stage marker of disease severity is still to be clarified.

## Introduction

COVID-19 is an infectious respiratory disease caused by the novel coronavirus, named as Severe Acute Respiratory Syndrome Coronavirus-2 (SARS-CoV-2)^1^. Briefly after COVID-19 outbreak in China, the disease spread around the world and the World Health Organization declared a pandemic^1^. The clinical presentation of COVID-19 can vary from asymptomatic to acute respiratory distress syndrome (ARDS) and respiratory insufficiency^2,3^. Most common underlying mechanism of respiratory failure in patients with COVID-19 is characterized by rapid activation of proinflammatory cytokines and inflammatory cell recruitment in the lungs (e. g. cytokine storm)^4,5^. An increased concentration of IL-6, TNF-α, IL-2, IL-8, IL-1β, G-CSF, IL-17, GM-CSF, IL10, MCP1, MIP1α, chemokines, galectins in serum, was detected in COVID-19 patients^6–8^. Interestingly, the role of immunosuppressive cytokines, especially IL-10 may also contribute to COVID-19 induced damage of the lungs in later stages of the disease^9^.

Galectins are oligosaccharides chain complexes located on the cell surface and play an important role in regulating immune reactions and inflammatory responses^10–12^. Gal-1 was the first identified protein in this family. Even though it is known that Gal-1 has many regulatory functions in viral infections, to date, the role of Gal-1 in immunopathogenesis of the COVID-19 disease has not been clarified. Study by Kazancioglu et al. investigated potential role of galectins in SARS-CoV-2 induced inflammation. The results imply that levels of Gal-1 and Galectin-3 (Gal-3) are elevated in COVID-19 patients in comparison to healthy patients^13^. The aim of this study was to determine the association of Gal-1 and innate immunity cytokines such as IL-1β, IL-6, IL-10, IL-23, IL-33 with disease severity, alongside with clinical, biochemical features and Chest X-rays (CXR) lung findings in 210 patients with COVID-19.

## Results

### Clinical features of COVID-19 patients

All of 210 patients included in the study met the criteria for COVID-19^14^ and were classified into the three groups according to disease severity. The median age of the patients in the mild group (54.9 ± 1.7) was significantly different from severe (59.5 ± 1.3) or critical one (61.3 ± 1.4) (Table 1). Among all of them, 135 patients (57.14%) were male. The analysis revealed a higher percentage of male patients and a lower percentage of female patients in group II and III with statistical significance (*p* = 0.041; Table 1). The most frequent auscultatory finding in group I was attenuated breathing sound in 44.3% of patients, with CXR findings described like interstitial thickening in 51.4%, focal consolidation in 28.5% and normal findings in 20% of patients. In group II, 58.5% of patients had auscultatory attenuated breathing sound, 48.5% audible crackles diffusely, with CXR findings described like multifocal consolidation in 44.2% of hospitalized patients, focal consolidation in 30% and interstitial thickening in 25.7%. In group III, 90% of patients had auscultatory audible crackles diffusely, attenuated breathing sound 85.7%, with diffuse alveolar changes/ARDS on CXR findings. Only 10 patients (14.2%) among this group had multifocal consolidation. Compared with mild group, the severe and critical groups were more likely to have dyspnea, fatigue, auscultatory audible crackles diffusely. The percentage of patients with these findings was significantly higher than in the mild group (*p* < 0.05; Table 1), which is in line with CXR findings of the patients. None of the patients in the severe and critical group had normal CRX finding, interstitial thickening or focal zones of consolidation (CRX I, II, III), and none of the patients in the mild group had multifocal zones of consolidation or diffuse alveolar changes/ARDS (CRX IV, V) (*p* = 0.000; Table 1).

**Table 1:** Demographics and clinical characteristics of patients with COVID-19.

	Total (n = 210)	Mild group (n = 70)	Severe group (n = 70)	Critical group (n = 70)	*p* value*
**Age mean ± SE**		54.9 ± 1.7	59.5 ± 1.3	61.3 ± 1.4	0.032
Sex					
Female	75 (42.6%)	30 (32.9%)	25 (28.6%)	20 (28.6%)	0.041
Male	135 (57.14%)	40 (64.2%)	45 (71.4%)	50 (71.4%)	0.041
**Clinical manifestations**					
Fever	182 (86.6%)	57 (81.4%)	60 (85.7%)	65 (92.8%)	> 0.05
Dry cough	153 (72.8%)	42 (60.0%)	51 (72.8%)	60 (85.7%)	> 0.05
Fatigue	130 (61.9%)	36 (51.4%)	45 (64.2%)	49 (70.0%)	0.016
Dyspnea	121(57.6%)	16 (22.8%)	35 (50.0%)	70 (100%)	0.043
Nausea and vomitung	67 (31.9%)	19 (27.1%)	21 (30%)	27 (38.5%)	> 0.05
Myalgia	47 (22.3%)	11 (15.7%)	13 (18.5%)	23 (32.8%)	> 0.05
Anosmia	24 (11.4%)	6 (8.5%)	9 (12.8%)	9 (12.8%)	> 0.05
Headache	79 (37.6%)	24 (34.2%)	26 (37%)	29 (41.4%)	> 0.05
Chest pain	15 (7.1%)	3 (4.2%)	6 (8.5%)	6 (8.5%)	> 0.05
Pharyngalgia	7 (3.3%)	4 (5.7%)	2 (0.9%)	1 (0.4%)	> 0.05
**Ausculattory findings**					
Normal	28 (13.3%)	22 (31.4%)	6 (8.5%)	0	0.000
Attenuated breathing sound	111 (52.8%)	31 (44.3%)	41 (58.5%)	39 (55.7%)	> 0.05
Sharpened respiratory sound	30 (14.2%)	5 (7.1%)	10 (14.2%)	15 (21.4%)	> 0.05
Audible cracks diffusly	124 (59.0%)	27 (38.5%)	34 (48.5%)	63 (90%)	0.012
Audible whistling	43 (20.4%)	2 (2.8%)	15 (21.4%)	26 (37.1%)	> 0.05
**CXR findings**					
Normal finding	14 (6.6%)	14 (20%)	0 (0%)	0 (0%)	0.000
Interstitial thickening	54 (25.7%)	36 (51.4%)	18 (25.7%)	0 (0%)	0.000
Focal consolidation	41 (19.5%)	20 (28.5%)	21 (30.0%)	0 (0%)	0.000
Multifocal consolidation	41 (19.5%)	0 (0%)	31 (44.2%)	10 (14.2%)	0.000
Diffuse alveolar changes—ARDS	60 (28.5%)	0 (0%)	0 (0%)	60 (85.7%)	0.000

Data expressed as mean, standard error (SR), frequency (percentage).

*p values indicate differences between mild, moderate and severe group *p* < 0. 05 was considered statistically significant.

In patients with severe and critical features, an increase in neutrophil count (*p* = 0.001), urea (*p* = 0.018), aspartate aminotransferase (AST) (*p* = 0.001), alanine aminotransferase (ALT), (*p* = 0. 001), direct bilirubin (DBIL) (*p* = 0.018), creatine kinase (CK) (*p* = 0.012), lactate dehydrogenase (LDH) (*p* = 0.001), D-dimer (*p* = 0.001), C-reactive protein (CRP), (*p* = 0.001), procalcitonin (PCT) (*p* = 0.001), Ferritin (*p* = 0.001) was noted, with reduced lymphocyte count (*p* = 0.001), monocyte count (*p* = 0.006), decreased levels of albumin (*p* = 0.001), lower values of oxygen saturation (Sa0_2_) (*p* = 0.001) and partial pressure of oxygen (p0_2_) (*p* = 0.001) (Table 2). There were no statistically significant differences in white blood cell count, erythrocytes and platelets, hemoglobin, levels of plasma glucose, creatinine, total bilirubin (TBIL), potassium (K^+^), sodium (Na^+^), iron (Fe), pH of blood, partial pressure of carbon dioxide (pC0_2_) (Table 2).

**Table 2:** Laboratory findings of patients with COVID-19.

Measured parametaras	Normal range	Mild	Moderate	Severe	*p* value*
White blood cell count, × 10^9^/L	3.7–10.0	6.6	8.1	8.9	> 0.05
Neutrophil count %	44.0–72.0	67.8%	77.3%	78.3%	0.001
Lymphocyte count %	20.0–46.0	21.0%	14.0%	12.9%	0.001
Monocyte count %	2.0–12.0	9.9%	8.3%	6.2%	0.001
Eritrocite count 10^12^/l	4.34–5.72	4.5	4.5	4.5	> 0.05
Trombocitecount 10^9^/l	135–450	223	237	248	> 0.05
Hemoglobin g/L	138–175	133	132	130	> 0.05
Glucose mmol/L	3.8–6.1	6.9	7.4	8.2	> 0.05
Urea mmol/L	3.0–8.0	6.5	6.7	8.2	0.018
Creatinin umol/L	49–106	95.5	90.6	93.2	> 0.05
BILTumol/L	0.0–21.0	10	10.9	11.7	> 0.05
BILDumol/L	0.0–6.6	2.6	3	3.6	0.018
AST U/L	0–40	38.1	52.8	59.7	0.001
ALT U/L	0–40	42	57	64	0.001
Albumin g/L	35–52	37.6	34.6	33.3	0.001
LDH U/L	220–450	510.0	618.3	830.5	0.001
CK U/L	0–171	167.4	215.0	247.0	0.012
D dimer ug/ml	< 0.50	1.3	2.2	3.2	0.001
CRP mg/L	0.0–5.0	87.6	92.4	134.7	0. 001
PCT ng/mL	0.5–2.0	0.2	0.6	1.1	0.001
p0_2_ kPa	10.7–13.3	10.4	7.8	6.6	0.001
pC0_2_ kPa	4.7–6.0	4.6	4.7	4.6	> 0.05
Sa02%	95–98	93.7	86.9	73.3	0.001
ph	7.35–7.45	7.47	7.45	7.45	> 0.05
K + mmol/L	3.5–4.5	3.9	3.8	3.7	> 0.05
Na + mmol/L	136–145	136.6	136.9	134.5	> 0.05
Fe umol/L	6.6–26	6.7	6.3	9.6	> 0.05
Feritin ug/L	20–300	510.9	894.3	1210.6	0.001

### Patients with more severe form of COVID-19 had increased systemic values of Gal-1

**Figure 1:**
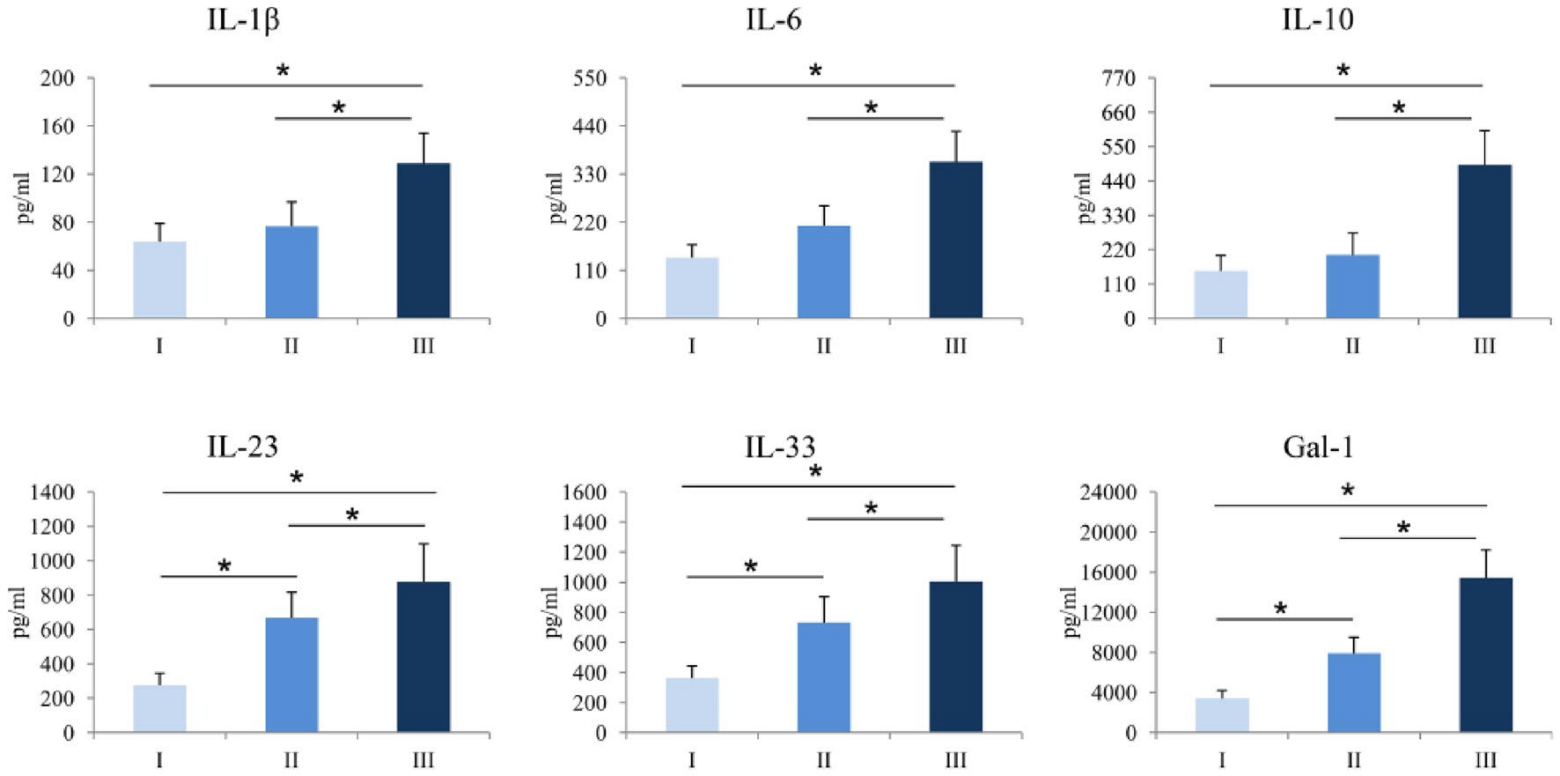
Serum values of pro-inflammatory and anti-inflammatory cytokines. Based on the disease severity, all COVID-19 patients were divided into three groups: I, II and III. Systemic levels of IL-1β, IL-6, IL-23, IL-33 and Gal-1 was measured by ELISA. Statistical significance was tested by Mann–Whitney Rank Sum test.

Serum pro- and anti-inflammatory cytokines of interest were measured in COVID-19 patients. Systemic values of IL-1β (*p* = 0.001), IL-6 (*p* = 0.001), IL-10 (*p* = 0.026), IL-23 (*p* = 0.001), IL-33 (*p* = 0.001), and Gal-1 (*p* = 0.001) were significantly higher in COVID-19 patients in stage III compared to patients in stage I and II (Fig. 1).

### Unchanged flow of ratios of Gal-1 and IL-10 with proinflammatory cytokines during COVID-19 development

**Figure 2:**
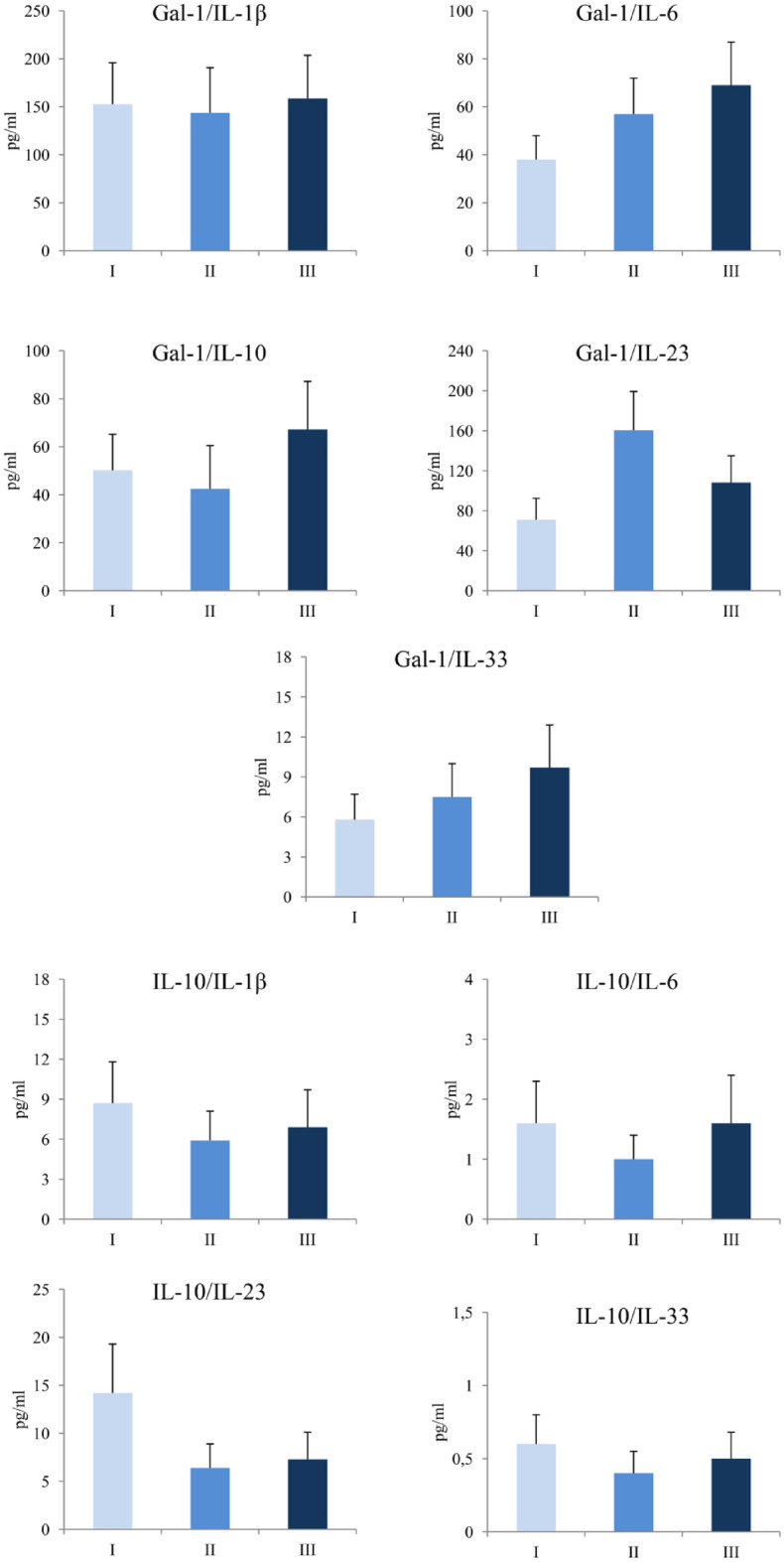
Ratio of Gal-1 and IL-10 with proinflammatory cytokines. Based on the disease severity, all COVID-19 patients were divided in three groups: I, II and III. IL-1β, IL-6, IL-23, IL-33 and Gal-1 was measured by ELISA. Ratios of Gal-1/IL-1β, Gal-1/IL-6, Gal-1/IL-23, Gal-1/IL-33, Gal-1/IL-10, IL-10/IL-1β, IL-10/IL-6, IL-10/IL-23 and IL-10/IL-33 were evaluated for each patient, separately. Statistical significance was tested by Mann–Whitney Rank Sum test.

Analyses of the ratios of Gal-1 and IL-10 with IL-1β, IL-6, IL-23 and IL-33 were done. There were no statistically significant differences in the ratios of Gal-1/IL-1β, Gal-1/IL-6, Gal-1/IL-23, Gal-1/IL-33 and Gal-1/IL-10 as well as IL-10/IL-1β, IL-10/IL-6, IL-10/IL-23, IL-10/IL-33 between defined groups of COVID-19 patients (Fig. 2).

### The strongest correlation between Gal-1 and pro- and anti-inflammatory cytokines in stage III of COVID-19

Analyses of correlation between Gal-1 and pro- and anti- inflammatory cytokines within stages of COVID-19 were made. In stage I of COVID-19, strong positive correlation was detected between Gal-1 and IL-33 (*p* = 0.00). Analyses of patients in stage II of COVID-19 revealed a moderate positive correlation between Gal-1 and IL-1β (*p* = 0.004) and strong positive correlations between Gal-1 and IL-23 (*p* = 0.001) and IL-33 (*p* = 0.001) (Table 3). In stage III of COVID-19 patients, moderate positive correlation was noticed between Gal-1 and IL-1β (*p* = 0.001) and IL6 (*p* = 0.005) and strong positive correlation between Gal-1 and IL-10 (*p* = 0.001), IL-23 (*p* = 0.001) and IL-33 (*p* = 0.001) (Table 3).

**Table 3:** Correlation between Gal-1 and pro- and anti-inflammatory cytokines.

Clin. stage	Gal-1					
	I		II		III	
	Pearson’s rho	*p* value	Pearson’s rho	*p* value	Pearson’s rho	*p* value
IL-1 β	0.089	0.511	**0.345**	**0.004**	**0.429**	**0.001**
IL-6	− 0.150	0.267	0.007	0.955	**0.374**	**0.005**
IL-10	− 0.121	0.369	0.082	0.509	**0.574**	**0.001**
IL-23	0.206	0.124	**0.603**	**0.001**	**0.656**	**0.001**
IL-33	**0.723**	**0.001**	**0.886**	**0.001**	**0.869**	**0.001**

Strength of correlation was defined as negative or positive weak (− 0.3 to − 0.1 or 0.1 to 0.3), moderate (− 0.5 to − 0.3 or 0.3 to 0.5), or strong (− 1.0 to − 0.5 or 0.5 to 1.0).

Statistically significance values (*p* < 0.05) was given in bold.

### Gal-1 correlates with clinical parameters of COVID-19

Analyses of correlation between Gal-1 and clinical parameters of COVID-19 were made. The results showed weak positive correlation between Gal-1 and dry cough (r = 0.242; *p* = 0.001) and headache (r = 0.158; *p* = 0.034), while weak negative correlation was detected between Gal-1 and normal breathing sound (r = 0.151; *p* = 0.043). Moderate positive correlation was detected between Gal-1 and chest radiographic findings (r = 0.352; *p* = 0.001).

Regression model for independent variables, including demographic features of the patients, clinical, biochemical parameters, serum levels of proinflammatory, anti-inflammatory cytokines and dependent variable (COVID-19 severity) showed significant results (Table 4). Advanced age and male sex are predictors for development of more severe form of COVID-19. Clinical manifestations such as dyspnea, fatigue and crackles are in positive linear association with COVID-19 severity. Analyses of laboratory findings also showed significant linear association with COVID-19 severity. Lymphocyte, monocyte count and albumin level were in negative linear association with COVID-19 severity. Parameters p0_2_ and Sa0_2_ were also in significant negative association with COVID-19 severity. Analyses of cytokines have shown that serum levels of IL-1β, IL-6, IL-10, IL-23, IL-33 and Gal-1 are in significant positive association with increased COVID-19 severity (Table 4).

**Table 4:** Linear regression analysis for dependent variable (COVID-19 severity).

	Unstandardized coefficients (*B*)	Std. error	Standardized coefficients (beta)	*t*	*p*
Age	0.009	0.004	0.172	2.531	0.012
Sex (Male)	0.302	0.124	0.166	2.433	0.016
Dyspnea	0.261	0.115	0.155	2.264	0.025
Fatigue	0.328	0.113	0.198	2.904	0.004
Crackles	0.322	0.109	0.200	2.950	0.004
Le	0.042	0.013	0.221	3.265	0.001
Neutrophil	0.015	0.004	0.284	4.231	0.001
Lymphocyte	− 0.020	0.005	− 0.284	− 4.214	0.001
Monocyte	− 0.042	0.010	− 0.278	− 4.129	0.001
Glucose	0.038	0.017	0.155	2.266	0.024
Urea	0.030	0.013	0.159	2.324	0.021
BILT	0.026	0.013	0.145	2.020	0.045
BILD	0.104	0.033	0.226	3.197	0.002
AST	0.005	0.001	0.226	3.324	0.001
ALT	0.003	0.001	0.177	2.604	0.010
Albumin	− 0.039	0.009	− 0.287	− 4.283	0.001
LDH	0.001	0.001	0.414	6.208	0.001
CRP	0.003	0.001	0.342	5.254	0.001
D dimer	0.049	0.019	0.180	2.648	0.009
PCT	0.276	0.093	0.200	2.958	0.003
p0_2_	− 0.247	0.021	− 0.634	− 11.848	0.001
Sa0_2_	− 0.103	0.011	− 0.552	− 9.547	0.001
Feritin	0.000330	0.000067	0.340	4.938	0.001
IL-1β	0.001	0.000462	0.199	2.739	0.007
Gal-1	0.00000709	0.0000025	0.207	2.828	0.005
IL-6	0.000446215	0.0001484	0.218	3.007	0.003
IL-10	0.00017607	0.00007588	0.169	2.320	0.021
IL-23	0.000158	0.0000524	0.218	3.015	0.003
IL-33	0.0001782	0.0000540	0.240	3.298	0.001

Examination of ROC curve demonstrates that Gal-1 can be a potential marker for stage II (sensitivity 89.5%, specificity 23.4%; cut off: 3959 pg/ml) and stage III detection (sensitivity 72.2%, specificity 25.9%; cut off: 9359 pg/ml) in COVID-19 patients.

## Discussion

In this study, we comprehensively analyzed the relationship between Gal-1 and innate immunity cytokine profile, clinical, biochemical outcomes, and radiographic findings with disease severity. This was a cross-sectional study with only one time point evaluation, which is a limitation of our research. All of 210 COVID 19 patients were classified into the mild, severe and critical group.

Critically ill patients were mostly male and older, compared to patients with mild to severe form of the disease, which indicate that gender and age correlated with disease severity. This was consistent with study by Hu et al.^15^ in which researchers suggested that age and gender may be indicators for poor outcome. In our study percentage of patients with dyspnea, fatigue, auscultatory audible crackles diffusely was significantly higher in severe and critical groups than in the mild one (Table 1).

Increased levels of neutrophil count, urea, AST, ALT, BILD, CK, LDH, D-dimer, CRP, PCT, Ferritin, reduced lymphocyte and monocyte count, decreased levels of albumin, and lower values of Sa0_2_ and p0_2_ are associated with the severity of COVID-19. These findings are also confirmed by linear regression analysis (Table 4). Our finding are in accordance with recent studies^16–18^. Significantly higher sera levels of PCT were observed in severe and critical group in comparison to the mild group. As PCT levels are increased in sepsis, systemic inflammatory response syndrome, bacterial, fungal, and parasitic infections, but not in viral infections, higher levels of this marker can suggest the possibility of multiple infections or cytokine storm in critically ill patients^19,20^. Increased levels of AST, ALT, DBIL, CK, LDH, D-dimer, suggested that SARS-CoV-2 infection may be associated with thrombosis or coagulopathy, myocardial injury, hepatic injury and other related organ damage^21,22^. Although higher values of white blood cell count, thrombocyte count, glucose, creatinine, Fe, BILT and lower values of hemoglobin, K^+^ and Na^+^ were detected in patients with severe and critical stage of COVID-19, statistical significance was not reached (Table 2). There was significant difference between CXR findings in mild, severe and critical group. In the mild group the most common radiographic finding was described as interstitial thickening (CXR II), in severe as multifocal zones of consolidation (CXR IV) and in critical group as diffuse alveolar damage/ARDS (CXR V). Less common CXR lung findings in the mild group were described like normal or focal zones of consolidation (CXR I, III), in severe like (CXR II, III), while in critical group, none of the patients had CRX I, II, III.

We analyzed systemic levels of IL-1β, IL-6, IL-10, IL-23, IL-33 and Gal-1. Gal-1, member of carbohydrate-binding protein family, is expressed in normal and in pathological cells and possesses variable biological functions^23^. The role of Gal-1 in progression of some type of viral infections is known. Bao et al. showed that in in vivo model, treatment with Gal-1 decreased viral titer and inflammatory cell infiltrations in lungs of mice infected with H1N1pdm09 virus, stimulates production of proinflammatory cytokines and chemokines in serum and bronchoalveolar lavage fluid^24^. During Influenza viral infection, level of Gal-1 was increased in lungs of infected mice, while Gal-1 knock-out mice were more prone to Influenza virus infection compared to wild-type group. Results with exogenous application of Gal-1 suggesting that Gal-1 acts in an antiviral way by tying up to viral surface and reduced its infectivity^25^. Also, it is confirmed that Gal-1 acts as adhesive molecule that enables attachment of HIV-1 virus to target cells thereby stimulating virus attachment and promoting infection^26^. There are no data regarding the effect of Galectin-1 on infectivity of SARS-CoV-2 virus. Our results showed that patients in the critical stage of disease—III, had the highest level of Gal-1 compared to patients in stage I and II (Fig. 1). These results are in line with previous studies suggesting that the level of Gal-1 increases during disease progression and stimulate viral progression.

To date, there are no data for use of Gal-1 inhibitors in COVID -19. Our results implicate that Gal-1 inhibitors may suppress the hyperimmune activation and be therapeutic approach in COVID-19 patients. We think that the use of Gal-1 inhibitors should be considered, however further investigations are necessary to evaluate their therapeutic potential.

It is known that Gal-1 can have opposite role in immune responses^23^. As immunostimulative molecule, previous study has shown that Gal-1 induced pro-inflammatory function of TNF-α thus facilitating inflammation in Sertoli cells^27^. On contrary, Motran et al. showed that Gal-1 stimulates apoptosis of Th1 cells and production of pro-Th2 cytokines^28^. Anti-inflammatory role of Gal-1 is reflected in the fact that it converted macrophages from their proinflammatory to anti-inflammatory profile thus terminating the inflammation^29^. Our previous work revealed predomination of Gal-1 over proinflammatory cytokines in patients with colorectal cancer, suggested on the immunomodulatory function of Gal-1 in the restriction of inflammation^30^. Our results confirmed the results of previous studies that systemic levels of proinflammatory cytokines IL-1 and IL-6 are rising during the progression of COVID-19 suggesting on ongoing development of inflammation^31^. We also found the strong positive correlation between Gal-1 and IL-33 (Table 3). IL-33 is a pleiotropic cytokine that functions as alarmin by stimulating Nuclear factor kappa-B, transcriptional factor, which further enhances the production of proinflammatory cytokines such as IL-1, IL-6 and promotes inflammation^32^. Gal–1–IL-33 axis may explain the subsequent correlation between Gal-1 and proinflammatory cytokines IL-1, IL-6 and IL-23 (Table 3). Kazancioglu et al. found that systemic level of Gal-1 is increased in patients with COVID-19 compared to healthy control^13^. In line with this result, we found out that level of Gal-1 increases during the infection and its value is the highest in patients in stage III (Fig. 1). Recent study showed positive association between Gal-1 and disease activity score in patients with Rheumatoid arthritis, potentiating Gal-1 as a disease prognostic marker^33^. In accordance with this finding, our results showed for the first time that Gal-1 positively correlated with clinical parameters of COVID-19 such as dry cough and headache, and chest radiographic findings. IL-10 is an immunosuppressive cytokine important for inhibition of functions of T cells, Natural killer cells and macrophages which are important for pathogen elimination, but also, are the cause of tissue damage^34^. Van der Leij et al. showed that Gal-1 realizes its immunosuppressive effect by stimulating production of IL-10^35^. We believe that in order to prevent and control progression of inflammation and tissue destruction as some kind of compensatory mechanism, immunosuppressive Gal-1 and consequently produced IL-10 increased in parallel with the increment of levels of proinflammatory cytokines, and moreover the relation of Gal-1 and proinflammatory cytokines didn’t change during COVID-19 progression (Figs. 1, 2). This assumption is confirmed not only by significant higher serum value of IL-10 in stage III of COVID-19 patients compared to stage I and II, but more importantly, by strong positive correlation that persists between Gal-1 and IL-10.

There are other galectins as Gal-3, Galectin-9 (Gal-9), known to be important contributing factors in cytokine release syndrome^8,36^. Although we did not observe these galectins in our study, many studies have shown significantly elevated levels of Gal-3 in sera of COVID-19 patients associated with worse outcomes and lower survival^8^. Also, in patients with severe form of COVID-19, elevated levels of Gal-9 was detected^36^.

Our data shows for the first time higher systemic values of Gal-1 in patients with severe COVID-19. The increment of IL-10 and Gal-1 as well as strong positive correlation between them in stage III of COVID-19 implicate on Gal-1 and IL-10 dependent immunomodulation. The precise mechanism of Gal-1 effect in COVID-19 and its potential as stage marker of disease severity is still to be clarified.

## Methods

### Ethical statement

This study was performed in the University Clinical Center of Kragujevac (Covid Center) and Faculty of Medical Sciences (Center for Molecular Medicine and Stem Cell Research), University of Kragujevac, Serbia. All examined subjects gave informed consent. Ethics Committees of the University Clinical Center of Kragujevac (Approval Number 01/20–406) and Faculty of Medical Sciences, University of Kragujevac (Approval Number 01–6776) have given ethical approvals. All research procedures were carried out in accordance with the Declaration of Helsinki and the Principle of Good Clinical Practice.

### Patients

This observational and cross-sectional study, from May 2020 to December 2020 included 210 confirmed cases of COVID-19. Real-time reverse transcription-PCR analysis were used for confirmation of COVID-19. 210 hospitalized COVID-19 patients were classified according to disease severity into the three groups: I—Mild group, consisting of 70 patients, with fever (37–38 °C), fatigue, pharyngalgia, anosmia, nausea, vomiting, headache, dry irritating cough, dyspnea, Sa0_2_ 91–100%, p0_2_ 8.5–13.3 kPa, with normal or weakened/sharpened respiratory noise; normal, interstitial thickening or focal zones of consolidation in CXR lung findings (CXR I, II, III); II—Severe group, consisting of 70 patients with fever of 38–39 °C, fatigue, frequent dry irritating cough, dyspnea, myalgia, nausea, vomiting, headache, chest pain, anosmia, Sa0_2_ 81–90%, p0_2_ 7.1–8.4 kPa, with auscultatory attenuated-inaudible respiratory noise, audible whistling/crackles in the lower segments of the lungs, with focal, multifocal zones of consolidation in CXR lung findings (CRX II, III, IV); III—Critical group, consisting of 70 patients with fever of 39–40 °C, frequent dry irritating cough, dyspnea, fatigue, myalgia, nausea, vomiting, headache, chest pain, Sa0_2_ ≤ 75–80%, p02 ≤ 5–7 kPa which require high frequency ventilation (HFV), non-invasive mechanical ventilation (Non Invasive Ventilation, NIV) or invasive mechanical ventilation, with auscultatory attenuated-inaudible respiratory noise, audible whistling/crackles diffusely, with multifocal zones of consolidation, diffuse alveolar changes/ARDS in CXR lung findings (CRX IV, V). Patients included in this study had not been previously treated with statins, corticosteroids, antibiotics, aminosalicylates, immunosuppressants, or any type of biologic therapy for at least two months before enrolling in the study.

### Data collection

Suspected COVID-19 patients had their nasopharyngeal swab samples taken and sent to designated authoritative laboratories to detect the SARS-CoV-2. Blood samples were drawn by venous puncture for blood cell counting, D-dimer, analyzes of biochemistry in plasma [urea, glycemia, creatinine, CRP, PCT, CK, AST, ALT, LDH, ALB, BILD, BILT, Fe, Ferritin, K^+^, Na^+^], and for immunoassay. Arterial blood gases (Sa0_2_, p0_2_, pC0_2_) were drawn and measured several times during the day by ion selective electrode on the automaton. For assessing lung damage, we used CXR digital portable anteroposterior (AP) technique. Radiographic features were described according to Yoon et al.^37^ following 5-point scoring scale: 1- normal; 2- patchy atelectasis and/or hyperinflation and/or bronchial wall thickening; 3- focal consolidation; 4- multifocal consolidation; and 5- diffuse alveolar changes.

### Measurement of cytokines levels in sera

As described in previous study, the blood was collected from patients at 8:00 am^38^. Sera was separated and stored at − 80 °C before use. For measurement the concentrations of IL-1β, IL-6, IL-10, IL-23, IL-33 and Gal-1 in serum samples, we used the commercially available ELISA tests following the instructions of the manufacturer (R&D Systems, Minneapolis, Minn, USA).

### Statistical analysis

Statistical Analysis Software IBM SPSS (version 23. 0) was used for performing all data analyses. The significance tests used for suitable purposes were Chi-square test, Student's t-test or Mann–Whitney U test. Data were shown as mean ± standard error of mean. Pearson’s or Spearman’s correlation assessed the possible relationship between variables. Strength of correlation was defined as negative or positive weak (− 0.3 to − 0.1 or 0.1 to 0.3), moderate (− 0.5 to − 0.3 or 0.3 to 0.5), or strong (− 1.0 to − 0.5 or 0.5 to 1.0). Also, basic linear regression model was used in order to identify possible predictors for disease severity. Statistical significance was set at *p* < 0.05.

### The limitations and strengths of study

Limitation of this study is cross sectional design. Due to absence of unified national platform for COVID-19 data consolidation, we only used confirmed cases from University Clinical Center of Kragujevac (Covid Center). Due to excluding criteria there is a possibility of selection bias, as another limitation of our study.

Strengths of our study is that it provides a detailed discussion about potential pathological role of Galectin-1 in COVID-19 patients. Well-defined inclusion criteria, data collection and statistical interpretation were done.
